# The Effect of Deoxyfluorination on Intermolecular Interactions in the Crystal Structures of 1,6-Anhydro-2,3-epimino-hexopyranoses

**DOI:** 10.3390/molecules27010278

**Published:** 2022-01-03

**Authors:** Martin Jakubec, Ivana Císařová, Jindřich Karban, Jan Sýkora

**Affiliations:** 1 Institute of Chemical Process Fundamentals of the Czech Academy of Sciences, v. v. i., Rozvojová 135, 165 02 Prague, Czech Republic; jakubecm@icpf.cas.cz (M.J.); karban@icpf.cas.cz (J.K.); 2 Department of Inorganic Chemistry, Faculty of Science, Charles University in Prague, Hlavova 2030, 128 40 Prague, Czech Republic; cisarova@natur.cuni.cz

**Keywords:** intermolecular interactions, deoxyfluorination, carbohydrates, fluorine interactions, X-ray crystallography, epimines

## Abstract

The effect of substitution on intermolecular interactions was investigated in a series of 1,6-anhydro-2,3-epimino-hexopyranoses. The study focused on the qualitative evaluation of intermolecular interactions using DFT calculations and the comparison of molecular arrangements in the crystal lattice. Altogether, ten crystal structures were compared, including two structures of C4-deoxygenated, four C4-deoxyfluorinated and four parent epimino pyranoses. It was found that the substitution of the original hydroxy group by hydrogen or fluorine leads to a weakening of the intermolecular interaction by approximately 4 kcal/mol. The strength of the intermolecular interactions was found to be in the following descending order: hydrogen bonding of hydroxy groups, hydrogen bonding of the amino group, interactions with fluorine and weak electrostatic interactions. The intermolecular interactions that involved fluorine atom were rather weak; however, they were often supported by other weak interactions. The fluorine atom was not able to substitute the role of the hydroxy group in molecular packing and the fluorine atoms interacted only weakly with the hydrogen atoms located at electropositive regions of the carbohydrate molecules. However, the fluorine interaction was not restricted to a single molecule but was spread over at least three other molecules. This feature is a base for similar molecule arrangements in the structures of related compounds, as we found for the C4-F*_ax_* and C4-F*_eq_* epimines presented here.

## 1. Introduction

Fluorinated carbohydrates are members of a large family of glycomimetics—chemically modified carbohydrates that structurally and functionally mimic natural carbohydrate ligands [1,2,3,4]. Fluorine features a similar van der Waals atomic radius and electronegativity to oxygen and thus can effectively mimic a hydroxyl group [5,6,7]. The replacement of a hydroxyl group by fluorine (deoxyfluorination) in a carbohydrate results in minimal steric alterations with respect to the parent molecule while affecting electron density, lipophilicity, glycosidic bond stability and other properties of the original carbohydrate molecule [8]. One of the most important changes is the loss of the hydrogen bond donating capacity and a significant weakening of the capacity to accept a hydrogen bond, which has consequences for molecular recognition. Nevertheless, epitopes based on fluorinated carbohydrate are structurally similar to natural epitopes. Deoxyfluorinated carbohydrates thus can serve as probes in studies focused on protein–carbohydrate interactions in which ^19^F NMR spectroscopy plays a prominent role [9,10,11]. On the other hand, deoxyfluorination can favorably influence the pharmacological properties of carbohydrate-based drugs, such as potency, lipophilicity, metabolic stability and resistance to the hydrolysis of the glycosidic bond [12,13,14,15,16,17,18,19,20].

The effect of deoxyfluorination on intermolecular interactions has been studied predominantly in solution, while similar studies in the solid state remain scarce [21,22]. In this paper, we focus on intermolecular interactions in the crystal structures of C4-deoxyfluorinated 1,6-anhydro-2,3-epimino-hexopyranoses and their comparison to the parent and deoxygenated molecules. These compounds contain an annulated aziridine (or epimine) ring, a three-membered saturated nitrogen heterocycle. The strained three-membered ring exhibits rich chemical reactivity; aziridine-containing compounds are therefore versatile synthetic intermediates and building blocks amenable to a variety of transformations [23,24,25]. Moreover, aziridine-containing compounds exhibit biological activity, mainly antitumoral [26]; some of them also occur naturally [27]. In the field of carbohydrate chemistry, pyranosides and furanosides with annulated aziridine rings have served mainly as intermediates in the synthesis of amino sugar analogues, the typical transformation being the nucleophilic opening of the three-membered ring [28,29]. In this respect, they function as nitrogen analogues of sugar epoxides. In this study of intermolecular interactions, the β-d-hexopyranose framework is rigidified by the 1,6-anhydro bridge and a 2,3-epimine functionality, generating a rigid tricyclic skeleton. The fluorine substitution takes place at C4 in the vicinity of the aziridine ring [30]. The effect of fluorine substitution on intermolecular interactions in crystal packing is evaluated through a comparison with the structures of the corresponding hydroxyl [31] and dihydrogen [32] analogues at the same C4 position, 10 crystal structures in total.

## 2. Experiments and Methods

### 2.1. Synthesis

The synthesis of C4-deoxyfluorinated epimines **II**, **III**, **VII** and **VIII** and C4-deoxy-epimines **I** and **VI** exploited aziridine ring closure through the reduction of vicinal trans azido sulfonates, as described previously [30,32]. The synthesis of *gulo*-epimine **V** relies on the aza-Payne rearrangement of vicinal *trans* azido epoxide [31]. *Allo*-epimine **IV** was obtained by cyclization under Mitsunobu conditions from a suitable 2-benzylamino derivative followed by hydrogenolytic N,O-debenzylation [33]. *Manno*-epimine **IX** was prepared through the reduction of a vicinal trans azido tosylate31 and hydrogenolytic *O*-debenzylation [34]. The synthesis of **X** is described in the Supplementary Materials.

### 2.2. DFT Calculation Details

For the calculation of the energies of the isolated molecules and their electrostatic potentials, the structures were optimized using the Gaussian09 [35] program, with B3LYP [36,37] as the functional and 6-311G(d,p)++ as a basis set. The vibrational analysis showed that all the structures correspond to the local minima in the potential energy surface. Avogadro software was used for the graphical presentation [38]. The energies of the respective pairs of molecules were obtained in the following manner. First, the coordinates of the selected pair of molecules were obtained from their X-ray structure and the hydrogens were allowed to relax using B3LYP/6-311G(d,p)++, while the coordinates of heavier atoms remained fixed. The optimized pair was used as a starting geometry for energy calculation, using B3LYP/6-311G(2df,2pd)++ and a D3 version of Grimme dispersion with Becke-Johnson damping [39], added with the keyword, empirical dispersion = gd3bj. The energies of all the pairs were then referenced to the pair with the lowest energy; the differences are shown in the following tables. Upon the reviewer’s request, the basis set superposition error (BSSE) [40] was analyzed using pairs of structure **III**. The obtained energy differences were found to be slightly lower; however, all the trends remained similar (for comparison, see Supplementary Materials). A detailed insight into the nature of the intermolecular interactions involved between molecules can be obtained through energy decomposition analysis (EDA) [41]; however, such an analysis was beyond the scope of this manuscript.

### 2.3. X-ray Crystallography

Single crystals suitable for X-ray crystallography were obtained from diethyl ether solution. In general, the compounds studied provided colorless crystalline material with a melting point within the range of 90–110 °C [30,31,32].

The diffraction data were collected on a Bruker D8 VENTURE Kappa Duo PHOTON 100 CMOS with monochromated Mo/Cu-Kα radiation. The structures were solved by direct methods (SHELXT [42]) and refined by full-matrix least-squares on F2 values (CRYSTALS [43]). All the heavy atoms were refined anisotropically. The hydrogen atoms were usually localized from the expected geometry and the difference electron density maps and were refined isotropically. ORTEP-3 [44] and a BIOVIA Discovery Studio Visualizer (Accelrys Inc., San Diego, CA, USA [45]) were used for the structure presentation.

The crystallographic data for the structures reported in this paper have been deposited with the Cambridge Crystallographic Data Centre as a supplementary publication. Copies of the data can be obtained free of charge on application to CCDC from the following e-mail address: deposit@ccdc.cam.ac.uk.

X-ray of 1,6-anhydro-2,3,4-trideoxy-2,3-epimino-β-d-*ribo*-hexopyranose **I**: C_6_H_9_NO_2_, M = 127.14 g/mol, orthorhombic system, space group *P2*_1_2_1_*2*, a = 9.3443(3), b = 7.9144(3), c = 8.0972(3) Å, Z = 4, V = 598.82(4) Å^3^, Dc = 1.41 g.cm^−3^, μ(Mo Kα) = 0.11 mm^−1^, T = 150 K, crystal dimensions of 0.28 × 0.42 × 0.55 mm. The final model converged to the final R = 0.0300 and R_w_ = 0.0740 using 1332 independent reflections (θ_max_ = 27.48°). CCDC registration number 2127197.

X-ray of 1,6-anhydro-2,3-dideoxy-2,3-epimino-β-d-allopyranose **IV**: C_6_H_9_NO_2_, M = 143.14 g/mol, orthorhombic system, space group *P2*_1_*2*_1_*2*_1_, a = 7.4175(2), b = 9.1635(2), c = 9.3760(2) Å, Z = 4, V = 637.29(3) Å^3^, Dc = 1.49 g.cm^−3^, μ(Mo Kα) = 0.12 mm^−1^, T = 150 K, crystal dimensions of 0.32 × 0.41 × 0.49 mm. The final model converged to the final R = 0.0256 and R_w_ = 0.0670 using 4762 independent reflections (θ_max_ = 29.99°). CCDC registration number 2127196.

X-ray of 1,6-anhydro-2,3-dideoxy-2,3-epimino-β-d-gulopyranose **V**: C_6_H_9_NO_2_, M = 143.14 g/mol, monoclinic system, space group *P2*_1_, a = 6.0539(10), b = 8.4214(16), c = 6.2298(12) Å, Z = 2, V = 306.43(10) Å^3^, Dc = 1.55 g.cm^−3^, μ(Cu Kα) = 1.06 mm^−1^, T = 150 K, crystal dimensions of 0.03 × 0.08 × 0.29 mm. Due to poor data, the hydrogen atoms were linked to the heavy atoms and were not refined. The final model converged to the final R = 0.0901 and R_w_ = 0.0240 using 773 independent reflections (θ_max_ = 69.38°). CCDC registration number 2127193.

X-ray of 1,6-anhydro-2,3,4-trideoxy-2,3-epimino-β-d-*lyxo*-hexopyranose **VI**: C_6_H_9_NO_2_, M = 127.14 g/mol, orthorhombic system, space group *P2*_1_*2*_1_*2*_1_, a = 6.5294(2), b = 7.5004(2), c = 23.9471(5) Å, Z = 8, V = 1172.76(5) Å^3^, Dc = 1.44 g.cm^−3^, μ(Mo Kα) = 0.11 mm^−1^, T = 150 K, crystal dimensions of 0.17 × 0.27 × 0.43 mm. The final model converged to the final R = 0.0296 and R_w_ = 0.0709 using 2567 independent reflections (θ_max_ = 27.38°). CCDC registration number 2127194.

X-ray of 1,6-anhydro-2,3-dideoxy-2,3-epimino-β-d-mannopyranose **IX**: C_6_H_9_NO_3_, M = 143.14 g/mol, orthorhombic system, space group *P2*_1_*2*_1_*2*_1_, a = 8.6099(2), b = 16.7111(5), c = 17.8865(5) Å, Z = 16, V = 2573.53(12) Å^3^, Dc = 1.48 g.cm^−3^, μ(Mo Kα) = 0.12 mm^−1^, T = 150 K, crystal dimensions of 0.52 × 0.63 × 0.70 mm. The final model converged to the final R = 0.0370 and R_w_ = 0.0877 using 5523 independent reflections (θ_max_ = 27.53°). CCDC registration number 2127198.

X-ray of 1,6-anhydro-2,3-dideoxy-2,3-epimino-β-d-talopyranose **X**: C_6_H_9_NO_2_, M = 143.14 g/mol, orthorhombic system, space group *P2*_1_*2*_1_*2*_1_, a = 7.4182(2), b = 8.9517(2), c = 9.5837(3) Å, Z = 4, V = 636.410(2) Å^3^, Dc = 1.49 g.cm^−3^, μ(Mo Kα) = 0.12 mm^−1^, T = 150 K, crystal dimensions of 0.17 × 0.33 × 0.50 mm. The final model converged to the final R = 0.0285 and R_w_ = 0.0694 using 1406 independent reflections (θ_max_ = 27.52°). CCDC registration number 2127195.

## 3. Results and Discussion

### 3.1. Nomenclature

**Scheme 1:**
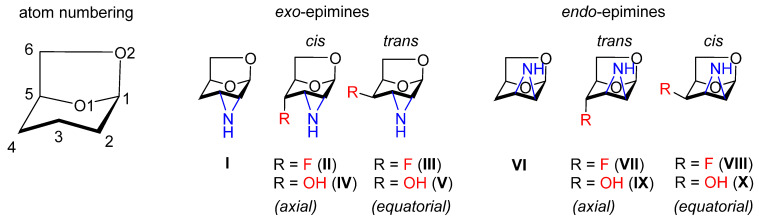
An overview of the series.

The six-membered pyranose ring of monosaccharides is relatively flexible, allowing the molecule to adopt various conformations. The most stable conformations are chair (C), boat (B) and twist (T), which can mutually interchange through less stable conformations such as half-chair (H), screw-boat (S) and envelope (E). The conformation can be fixed through derivatization or the attachment of an additional ring. In the case of this study, it is formal dehydration that bridges carbon atoms C1 and C6 (forming a five-membered 1,3-dioxolane ring), and the annulation of an aziridine (or epimine) ring at C2 and C3. This transformation fixes the six-membered tetrahydropyran ring in an envelope conformation with the pyranose oxygen distorted from the plane defined by carbons C1–C5 [30]. In this molecule, all the carbon atoms are used to fix the conformation except for carbon C4, which can be used for further modification and the subsequent study of the resulting substitution effect. In this paper, the 1,6-anhydrohexopyranoses containing an annulated 2,3-aziridine ring **IV**, **V**, **IX**, and **X** (Scheme 1) were modified at C4 by deoxygenation (giving rise to compounds **I** and **VI**) or deoxyfluorination (leading to compounds **II**, **III**, **VII** and **VIII**). A combination of an *exo*- and *endo*-epimine arrangements [46] with respect to the bicyclic 6,8-dioxabicyclo-[3,2,1]-octane skeleton, together with a substitution to an axial (ax) or equatorial (eq) position at C4, afforded a series of ten structures; five *exo*- and five *endo*- 2,3-epimino derivatives of 1,6-anhydro-β-d-hexopyranoses (Scheme 1). The designations *cis* (compounds **II**, **IV**, **VIII** and **X**) and *trans* (compounds **III**, **V**, **VII** and **IX**) refer to the relationship between the aziridine ring and the C4 substituent. Compounds **I**, **IV**, **V**, **VI**, **IX** and **X** were prepared according to known procedures (see Experimental Section). Single crystals suitable for X-ray crystallography were obtained by crystallization from diethyl ether. The four crystal structures containing C4-fluorinated epimines **II**, **III**, **VII** and **VIII** were published previously [30] (CSD refcodes GUZPAT, GUZNUL, GUZPEX and GUZPIB, respectively). The crystal structures served as a base for study of the substitution effect on the molecular arrangement in the crystal lattice.

### 3.2. Options for the Formation of Intermolecular Interactions

**Figure 1:**
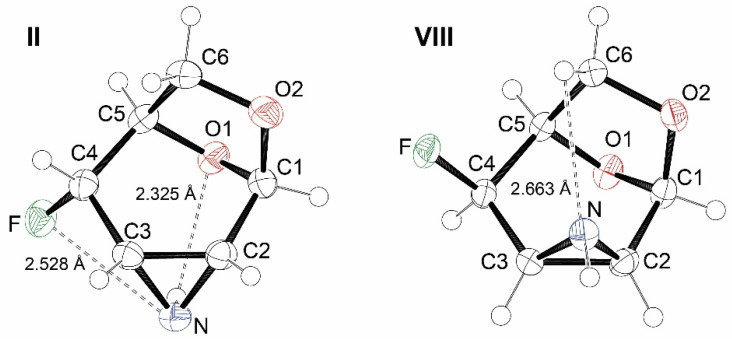
ORTEP presentation of structure **II** (**left**) and **VIII** (**right**) showing the intramolecular stabilization of NH.

Generally, the molecules in the series can utilize two basic types of intermolecular interaction: hydrogen bonding and van der Waals, or weaker electrostatic interactions. Hydrogen bonding utilizes both oxygen atoms (pyranose oxygen O1 and 1,6-anhydro oxygen O2, see Scheme 1 or Figure 1 for numbering) as hydrogen bond acceptors. As these atoms are tightly fixed in the rigid tricyclic skeleton, their electron lone pairs point in the same directions in all the molecules studied. Another atom functioning as an acceptor of hydrogen bond is the nitrogen of the epimine group. The nitrogen lone pair can point inside or outside of the molecule as nitrogen can easily undergo a pyramidal inversion and switch the orientation of its lone pair with hydrogen. The DFT calculations indicated that in most cases, the orientation of the hydrogen inside the molecule was more favorable due to the stabilization via intramolecular N-H···O1/O2 hydrogen bond. The energy difference was much higher when the fluorine or hydroxy groups were in the *cis* arrangement to the epimine group. In general, the energy difference was significantly higher for the *exo*-epimines than for the *endo*-epimines (Table 1).

The intramolecular NH···O1 hydrogen bond was found in three cases of *exo*-epimine crystal structures (**II**, **IV** and **V**); two of them possessed a *cis* arrangement of the fluorine or hydroxy group to the epimine (**II** and **IV**). The hydrogen atom of the epimine was involved in the intramolecular hydrogen bonding, mainly towards pyranose oxygen O1 (Figure 1). In these structures, the hydrogen atom was disqualified from participation in intermolecular interactions in the molecular packing. The epimine group could then operate only as an acceptor of hydrogen bond via nitrogen lone pair. In the rest of the *exo*-epimine structures (**I** and **III**), NH was found as an important donor of hydrogen bond. The formation of the intramolecular hydrogen bond of the epimine group in *endo*-epimines was also observed in solution by IR spectroscopy. However, in all the solid-state structures presented here, the lone nitrogen electron pair of *endo*-epimines was oriented towards C6, where it was involved in a weak interaction with the endo C6 hydrogen (Figure 1).

**Figure 2:**
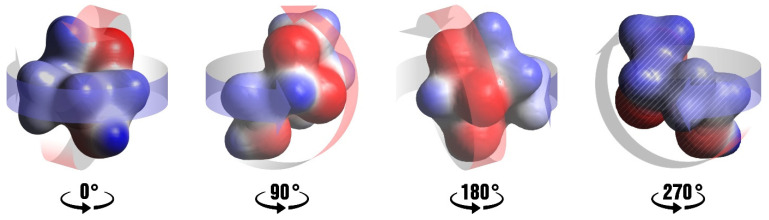
An electrostatic potential mapped on the surface of **I**; electro-positive regions in blue, electro-negative regions in red.

The deoxyfluorination at C4 introduced fluorine as another hydrogen bond acceptor with non-specific directionality, while the C4 hydroxy group could act as a simultaneous donor and acceptor of hydrogen bonding. Contrary to the fixed orientation of the NH group, the OH group could freely rotate around the C4-O bond and adopt any orientation, depending on which was the most favorable for the formation of a hydrogen bond. The structures containing two hydrogen atoms at C4 could utilize these atoms only in weak electrostatic interactions as they were located in the electro-positively charged part of the molecule. The electro-positive region passed through all hydrogen atoms located on the carbon skeleton. Apart from C6, all the carbon atoms were located in the equatorial part of the molecule and created a strip from C1 to C5, where it was interrupted by O1 (Figure 2). By contrast, the electro-negatively charged atoms were located on a vertical axis, represented mainly by O1 and O2. The location of the formed electronegative strip depended on the orientation of the epimine group; it was partially located at the lower or upper part of the molecule for the *exo*-epimines and *endo*-epimines, respectively. Further extension of the electronegative region was provided by fluorine and hydroxy substituents, depending on their particular placement.

### 3.3. The Strongest Intermolecular Interactions Found in **I**–**X**

**Figure 3:**
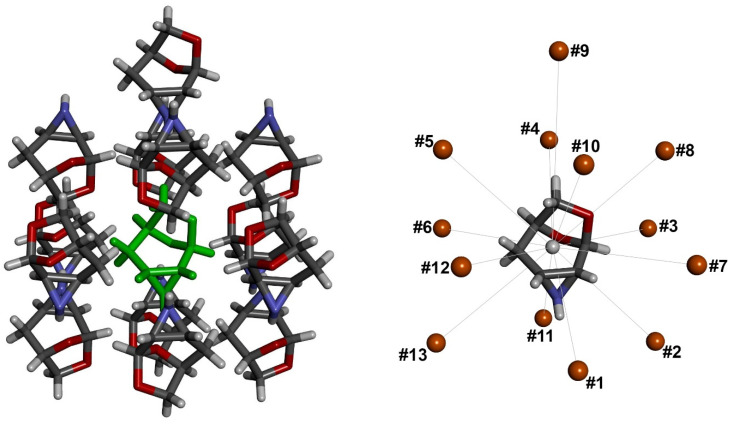
Molecular packing in **I** (**left**) and coding of the surrounding molecules expressed as molecular centroids (**right**).

The above-mentioned types of intermolecular interaction were found in molecules surrounding the parent molecule in the crystal lattice. In general, the number of surrounding molecules varied depending on the size, shape and chemical nature of the molecule. In the structures of the epimines studied, the number varied from 12 to 14, with six molecules forming an equatorial ring surrounding the parent molecule from C1 to O1 and three or four molecules above and below approaching the molecule from the top (C6 and O2) and bottom, respectively (Figure 3). The surrounding molecules were generated from the parent molecule by a symmetry operation or translation.

**Figure 4:**
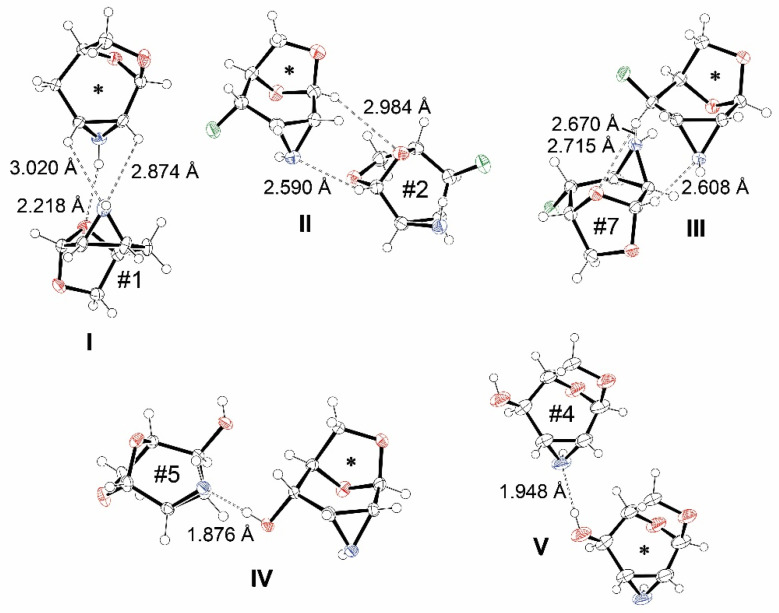
ORTEP presentation of the strongest intermolecular interactions found in the structures of *exo*-epimines **I**–**V**. * denotes a parent molecule.

**Figure 5:**
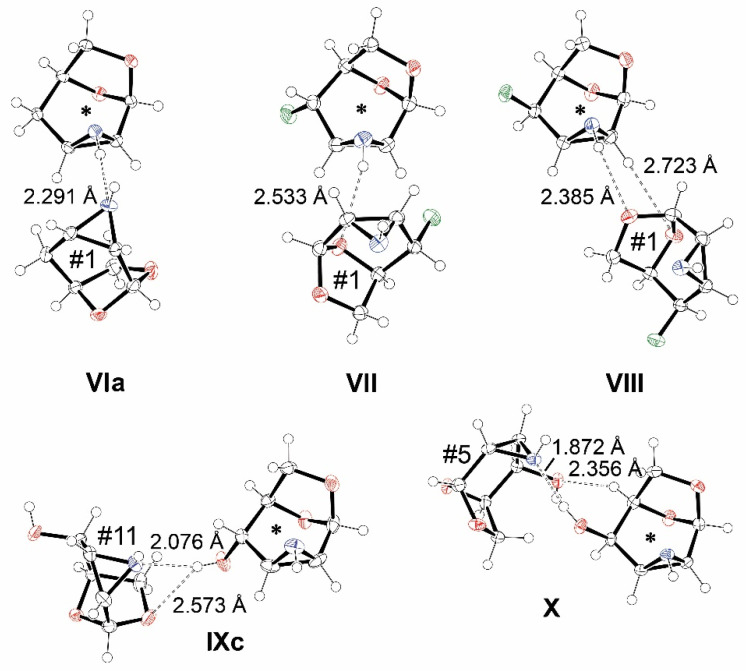
ORTEP presentation of the strongest intermolecular interactions found in the structures of *endo*-epimines **VI**–**X**. * denotes a parent molecule.

The strength of individual interactions was estimated via DFT calculations, where the energy of each pair of interacting molecules was calculated and mutually compared within a single crystal structure. This analysis identified the strongest interaction and provided an overall energy range of interacting pairs in a given crystal lattice. The complete list of DFT calculation results including the numbering of molecular pairs in each structure can be found in the Supplementary Materials. The intermolecular interaction of NH with O1 serving as an acceptor of hydrogen bond was found in almost all C4 deoxygenated and C4 deoxyfluorinated epimine derivatives. In these structures, the energy difference between the strongest and weakest interaction in given crystal lattices ranged from 4.20 to 5.07 kcal/mol. The only exception was **II,** which is discussed below in detail. In the structures of the hydroxy epimines, interactions involving the hydroxy group were found to be the strongest. The nitrogen atom usually acted as a hydrogen bond acceptor via the interaction of its lone pair. The energy difference ranged in hydroxy epimines from 8.20 to 9.11 kcal/mol, indicating the formation of much stronger intermolecular interactions than in the rest of the structures. The smallest energy difference between molecular interactions was found in the structure of *exo*-epimine **II**, where NH was involved in intramolecular interaction with fluorine and pyranose oxygen. The strongest interaction found in the molecular packing of **II** was the interaction between the electron-poor and -rich regions of two adjacent molecules, which was followed by other weak interactions also involving the fluorine atom. The strongest interactions found in the structures of the *exo*- and *endo*-epimines can be seen in Figure 4 and Figure 5, respectively.

In general, the fluorine atom did not act as an acceptor of hydrogen bonds donated by the OH or NH group in the structures studied. The fluorine atom was mainly involved in weaker interactions with hydrogen atoms localized on the carbon skeleton. The interaction of fluorine in combination with other weak interactions could lead to rather strong associations with the parent molecule. In **II**, the fluorine interaction with hydrogen at C2 of the epimine was supported by the interaction of C2 and C6 hydrogen with nitrogen and pyranose oxygen (ΔE = 1.16 kcal/mol, molecule no. 9). In **VII**, fluorine interacted with C4 hydrogen of molecule no. 9, which interacted reversely by its fluorine with C3 hydrogen. This double fluorine interaction of two molecules featured ΔE = 1.60 kcal/mol. In **VIII**, fluorine interaction with C5 hydrogen was supported by the interaction of C4 with the nitrogen lone pair (ΔE = 0.90 kcal/mol, molecule no. 10). In **III**, the fluorine interaction was not specific. The closest hydrogen was located at C2 of molecule no. 5 without any other support, resulting in one of the weakest interactions in the structure (ΔE = 4.95 kcal/mol). However, it is worth mentioning that the interaction of the fluorine atom was not restricted to one molecule but interacted weakly with two or three other molecules in the surroundings and contributed significantly to the molecular arrangement in the surroundings of the central molecule.

### 3.4. Comparison of Molecular Arrangements in Structures **VI** and **IX** Containing More Independent Molecules

**Figure 6:**
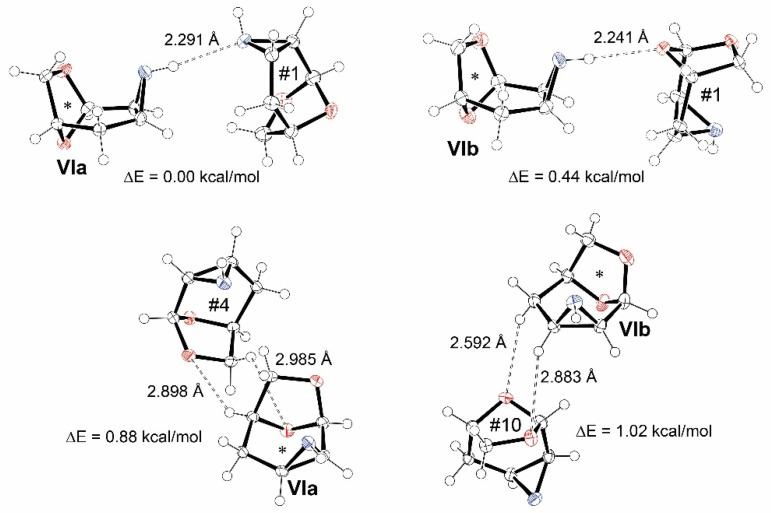
ORTEP presentation of the strongest intermolecular interactions found in **VI**. * denotes a parent molecule.

The substitution effect on the formation of intermolecular interactions was inspected through the mutual comparison of molecular packing in the epimine structures. The role of the epimine group was assessed through a separated comparison of *exo* and *endo* structures; similar motifs in crystal packing should be found among structures with the same substitution or, alternatively, some features that are independent of the substitution could also be revealed across all the structures. In addition to the mutual comparison of all the structures, the structures containing more molecules in the independent part of the unit cell provided unique material for the detailed assessment of the capabilities of a given molecule for the formation of intermolecular interactions. In particular, the structure of C4 deoxygenated *endo*-epimine **VI** contained two independent molecules, denoted as **VIa** and **VIb**, while the structure of C4-OH_ax_ *endo*-epimine **IX** contained four molecules (**IXa**–**d**). It is very likely that each independent molecule would somehow utilize different intermolecular interactions despite possessing the same or similar conformation. In the case of **VI**, the independent molecules **VIa** and **VIb** were each surrounded by six molecules of **VIa** and six molecules of **VIb**, but no obvious similarities were apparent in the molecular arrangement around the parent molecule. Both molecules formed the strongest interaction via the hydrogen bonding of their NH group. While in **VIa**, NH interacted with nitrogen lone pair of an adjacent molecule, in **VIb**, NH interacted with a lone pair of pyranose oxygen. The latter interaction was 0.4 kcal/mol weaker. The rest of the intermolecular interactions were provided by weak electrostatic interactions. However, a combination of two such weak interactions could lead to the formation of a quite strong interaction between two molecules with an energy difference of around 1 kcal/mol when compared to the strongest interaction found in the structure. An overview of the strongest intermolecular interactions found in **VI** can be seen in Figure 6.

**Figure 7:**
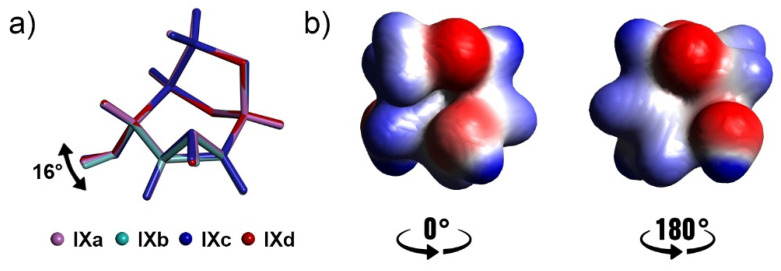
Overlaid independent molecules **IX** (**a**); electrostatic potential mapped on the surface of **IX** (**b**), top view (**left**), bottom view (**right**).

**Figure 8:**
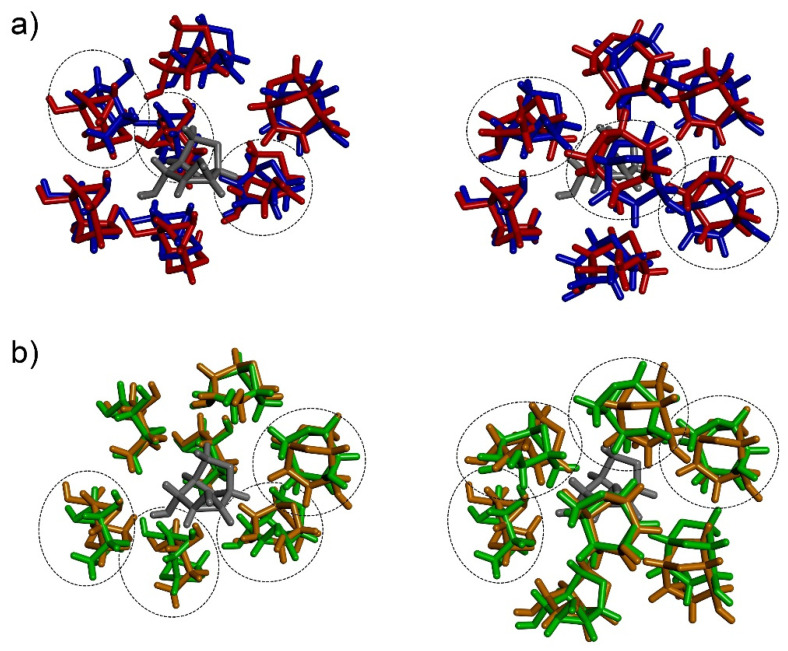
An overlay of the molecules surrounding **IXa** (blue) and **IXb** (red) parent molecule (**a**), and **IXc** (orange) and **IXd** (green) parent molecule (**b**); molecules in the back (left) and in the front (right). Parent molecules are depicted in gray. Dashed circles indicate the molecules mutually rotated by 180° with the switched OH and NH interaction.

As mentioned above, the independent part of the unit cell in the structure of **IX** was formed by four molecules. The geometries of all four molecules were practically identical, including the orientation of the hydroxy group, which differed among molecules **IXa**–**d** by only 16° (Figure 7a). Each independent molecule was surrounded by 12 molecules; for **IXa** and also for **IXd** it was 2(**IXa**)-4(**IXb**)-4(**IXc**)-2(**IXd**), for **IXb** 4-0-4-4, and 4-4-0-4 for **IXc** (see Supplementary Materials). The strongest interactions found in the structure involved the hydroxy group. In the case of **IXa**–**c**, the nitrogen lone pair served as an acceptor of hydrogen bond from the OH group, while in **IXd**, OH from **IXc** was the acceptor. The NH group as a donor of hydrogen bonds provided slightly weaker interactions. The acceptors of this interaction were NH in **IXa**, OH in **IXb** and O1 in **IXc**,**d** (Table 2). An interesting feature of **IX** was the mutual position of OH and NH groups in the molecule. It seems that both groups could be interchanged (by rotation of the molecule), while the ability of the molecule to participate in weaker interactions remained almost unaffected as the original electro-negative/positive regions were replaced by electro-negative/positive regions from the opposite side of the molecule (Figure 7b). This feature was apparent when the molecular packing around **IXa** was compared to that of **IXb**. The only difference was in the acceptor of the hydrogen bond from the NH group (Table 2), which was caused precisely by the exchange of OH and NH in intermolecular interactions. Otherwise, the molecules surrounding the parent molecules **IXa** and **IXb** were in similar positions, utilizing similar interactions (Figure 8a). Similarly to **IXa** and **IXb**, the hydrogen bonds donated by the OH group were also accepted in **IXc** by the nitrogen lone pair of another molecule. On the other hand, this was the only interaction common to all three molecules; the rest of the interactions were of a different origin in **IXc**. The molecules surrounding **IXc** were in similar positions, as in **IXa** and **IXb**, but a similar packing motif was found only for molecules tied by OH···NH interactions. As a parent molecule, **IXd** accepted hydrogen bonds from OH by its NH group, similarly to **IXa** and **IXb**. However, the greatest number of similarities in molecular packing was found for **IXd** and **IXc**. Furthermore, in this case, the OH group was exchanged in some interactions by NH, while the arrangement of the other molecules remained the same (Figure 8b).

### 3.5. Comparison of Molecular Arrangements in Exo-Epimines **I**–**V** and in Endo-Epimines **VI**–**X**

The comparison of packing motifs within the group of *exo*- or *endo*-epimines should reflect the role of common molecular fragments i.e., the aziridine group, the carbon skeleton, bridging oxygen atoms. The question is whether the effect of these fragments is preserved in the presence of a different substitution.

**Figure 9:**
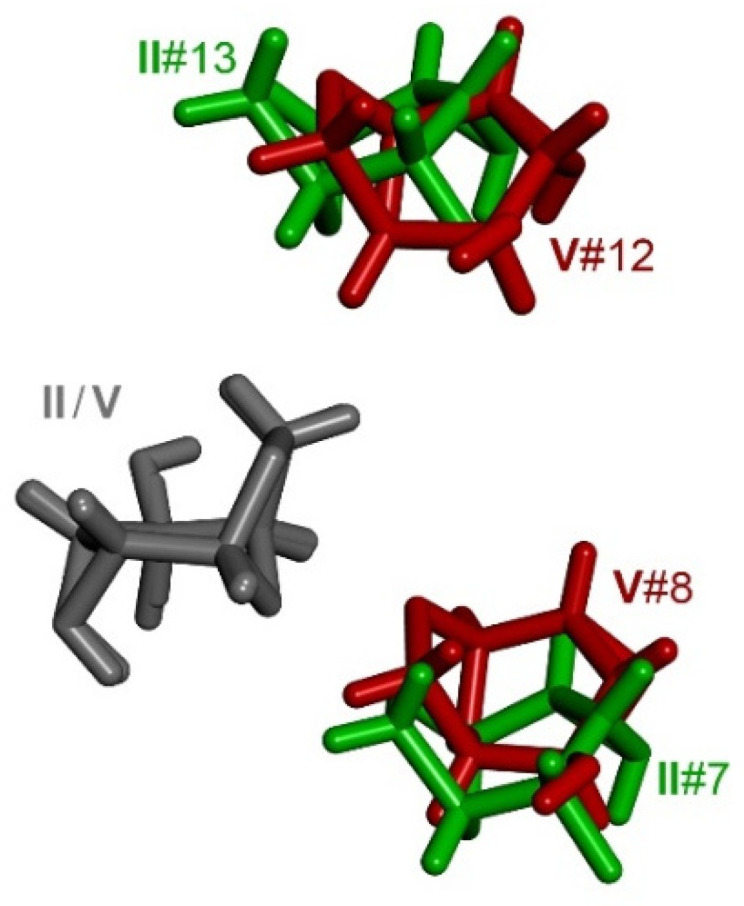
An overlay of similar segments in molecular packing in **II** (green) and **V** (red). Parent molecules are depicted in gray.

In the crystal structures of exo-epimines **I**–**V**, the intramolecular hydrogen bonding of the epimine moiety was preserved in three of them. This fact, in combination with varying substitution at C4, resulted in different molecular packing; a thorough analysis did not show any common intermolecular interaction. The only similarity was found in weak interactions in structure **II** and **V**, where hydrogen atoms at C6 approached the pyranose oxygen O1 and hydrogen atoms at C5 approached the anhydro oxygen O2. In both structures, two molecules in the surroundings of the parent molecule served alternately as donors and acceptors of these weak interactions. Both molecules were coupled with the parent molecule by the same symmetry operation (Figure 9). The same nature of interactions binding both molecules to the parent molecule was documented by the same energy difference calculated by DFT (see Supplementary Materials).

**Figure 10:**
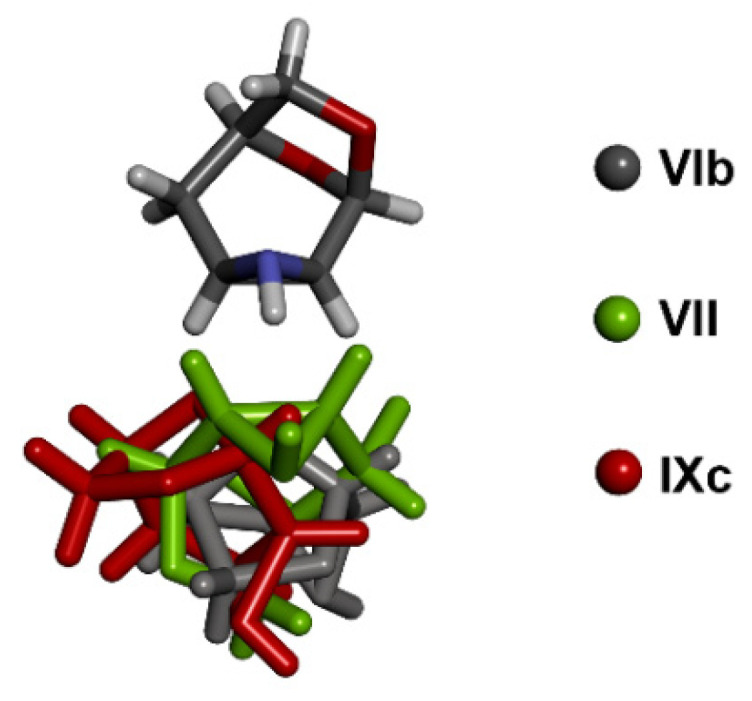
Different orientations of adjacent molecules utilizing hydrogen bonding of NH towards pyranose oxygen O1 in structures **VI** (gray), **VII** (green) and **IX** (red).

Contrary to the *exo*-epimines, the *end*o-epimines **VI**–**X** did not form an intramolecular hydrogen bond; thus, the configuration of the nitrogen atom was the same in all the structures studied in this group. The analysis of the intermolecular interactions in five *endo*-epimine structures was based on a comparison of nine molecular packings as a consequence of the presence of two and four independent molecules in the crystals of **VI** and **IX**, respectively, as discussed above. The intermolecular hydrogen bonding of NH towards pyranose oxygen O1 was found in all the structures. This interaction was the strongest in the unsubstituted and fluorine-substituted derivatives, while in hydroxy derivatives it played only a supportive role and the strongest interaction involved primarily the OH group. Surprisingly, no common motif was found in the structures based on NH···O1 interactions as the molecules involved possessed different mutual orientations (Figure 10).

Different arrangements of adjacent molecules utilizing NH hydrogen bonding together with the interactions of different substituents led to different molecular assembly in these structures. Nevertheless, certain similarities were found in the crystal packing of some *endo*-epimines; these can be attributed to weaker interactions. A detailed discussion of the similar packing motifs found in *endo*-epimines can be found in the Supplementary Materials.

### 3.6. Comparison of Molecular Arrangements in the Ax- and Eq-Substituted Epimines

A comparison of the structures with identical substituents in the same orientation, axial or equatorial, can reveal whether the substituent determines the arrangement of the molecules in the crystal lattice regardless of the orientation of the aziridine group.

**Figure 11:**
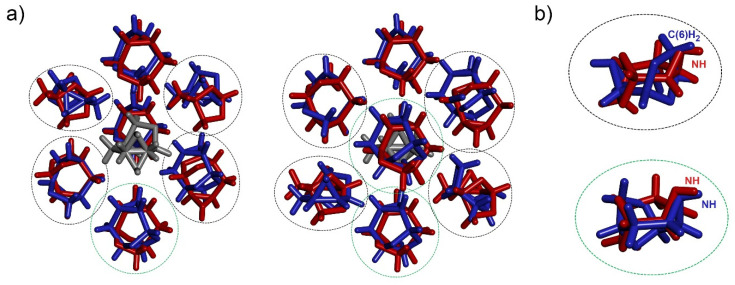
An overlay of molecular arrangements around **I** (red) and **VIa** (blue), molecules in the back (**left**) and in the front (**right**). Parent molecules are depicted in gray (**a**). Dashed circles indicate different orientation of the molecules switching in interaction CH_2_ and NH group (black) and positioning NH group to the same location (green). Detail of the different orientation of the molecules (**b**).

In the structures of the deoxygenated epimines **I** and **VI**, namely **VIa**, the molecules in the surroundings of the parent molecule were in practically the same position, although the strongest interaction was slightly different in both structures. In both cases, the strongest interaction involved the NH group as a donor of the hydrogen bond. While in **I**, the acceptor of this hydrogen bond was pyranose oxygen O1, in **VIa**, it was a nitrogen lone pair. Interestingly, both molecules, which employed strong interactions, were in the same spatial position in both structures, differing only in orientation. This feature enabled the placing of the rest of the molecules in the same position, as well as applying similar interactions to the parent molecule. A certain portion of the molecules utilized exactly the same interactions; these molecules also possessed a similar orientation towards the parent molecule in both structures. The other molecules were in different orientations, which was convenient for the formation of intermolecular interactions (Figure 11). The mutual rotation of the molecules in the same positions compensated to a certain degree for the different orientation of the NH group in the *exo*- and *endo*-epimines. It is worth noting that when rotated by 180°, a single molecule of **I** possesses a similar distribution of electrostatic potential on the surface as found in molecule **VI** merely by replacing NH group by CH_2_. This feature probably contributed significantly to the similarity found in both molecular arrangements. On the other hand, no similarities were found for **I** and **VIb**. Obviously, the orientation of NH did not determine the molecular arrangement around **VIa**, while for **VIb** it did.

**Figure 12:**
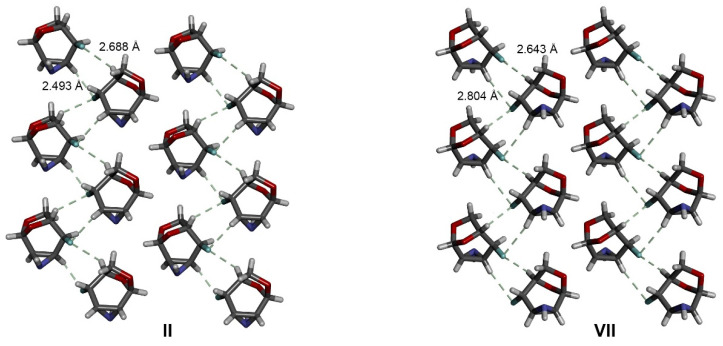
Interaction of fluorine atoms in the molecular packing of **II** (**left**) and **VII** (**right**).

**Figure 13:**
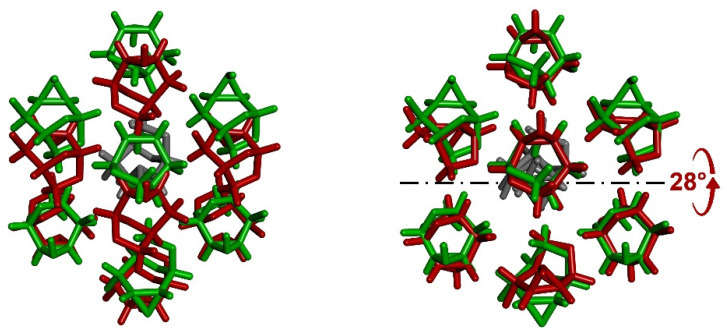
Front view (**left**) of overlaid structures **II** (green) and **VII** (red), molecules in the back (**left**) and in the front (**right**). Front view (**right**) of overlaid structures **II** (green) and **VII** (red) where **VII** is rotated approximately by 28°. Parent molecules are depicted in gray.

The association of the molecules in the structures of C4-axial fluoro epimines **II** and **VII** appeared different at first sight as only the molecules on the side of the parent molecule seemed to be at least in similar positions. Nevertheless, both structures utilized similar intermolecular interactions involving fluorine atoms. In both structures, the mutual interaction of the substituents at C4 gave rise to molecular chains based primarily on the interaction between hydrogen at C3 and C5 and the fluorine atom of the adjacent molecule. In a simplified way, the interactions within one chain can be described as ···HCCF···HCCF···HCCF··· (Figure 12). When the structures were aligned in the chain direction, all the molecules fitted almost perfectly to their counterparts in the other structure, except for the parent molecules, which were then mutually rotated by approximately 28° around the horizontal axis (Figure 13). The identical molecular assembly in both structures manifested the effect of fluorine atom, which did not form a strong interaction with a single molecule but interacted weakly with at least three other molecules in the proximate surroundings. This feature determined the molecular arrangement in the whole structure, even though there were stronger interactions present.

**Figure 14:**
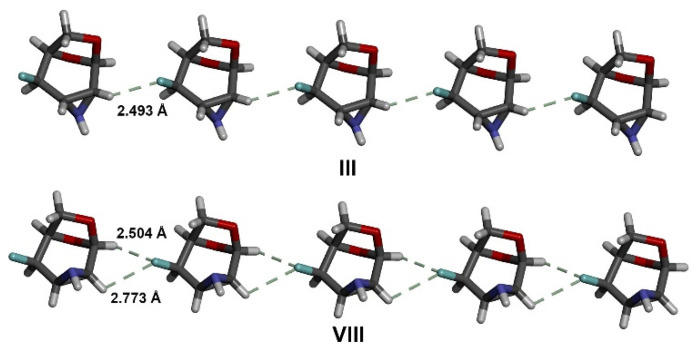
A chain formation in the structures **III** (**top**) and **VIII** (**bottom**).

In the structures of C4-equatorial fluoro epimines **III** and **VIII**, a similar arrangement was found only for two molecules approaching the parent molecule from the side. Interacting molecules utilized similar interactions of fluorine atoms with hydrogen at C2 (in **VIII**, it is supported by interaction with hydrogen at C1). These molecules were generated by translation of the parent molecule and the fluorine interaction was not supported by any other interaction. This arrangement was tied together by the weakest interaction in both structures (molecules nos. 2 and 5, see Figure 14 and Supplementary Materials). However, the same orientation of the fluorine yielded a similar molecule arrangement in its vicinity in both structures, despite the formal weakness of the interaction, as suggested by the DFT calculations.

**Figure 15:**
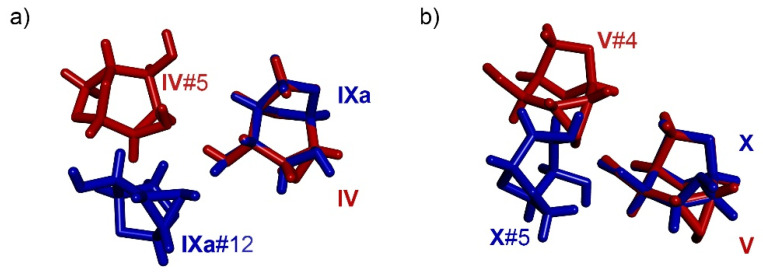
The intermolecular interaction of the OH group in the C4-OH_ax_ epimines (**a**) and the C4-OH_eq_ epimines (**b**).

A comparison of the unmodified carbohydrates with the same orientation of the C4 hydroxy group, axial **IV** and **IX**, and equatorial **V** and **X** did not reveal any common features in their molecular arrangements. In these four structures, hydrogen bonding of OH as the strongest interaction played a dominant role in the molecule arrangement. The involvement of NH in the intramolecular hydrogen bond in both *exo*-epimine structures **IV** and **V** was probably the main reason for the completely different packing of both groups. As discussed above, in **IV** and **V**, the NH group served only as an acceptor of intermolecular hydrogen bonds, while in **IX** and **X** it served as both donor and acceptor. The most favorable molecule arrangement found in the crystal lattice of C4-OH_ax_ epimines **IV** and **IX** directed the O-H bond to different directions (Figure 15a). The C4-OH_eq_ epimines **V** and **X** utilized OH···NH in the same direction; however, the interacting molecules approached the parent molecule from a different direction (Figure 15b), which can also be seen as a consequence of the different orientation of the NH group.

## 4. Conclusions

The effect of substitution on intermolecular interactions was investigated in a series of 1,6-anhydro-2,3-epimino-hexopyranoses. The strength of intermolecular interactions was assessed using DFT calculations, providing the background for a detailed comparison of the molecular arrangements in ten crystal structures, which included two structures of C4-deoxygenated, four C4-deoxyfluorinated and four parent carbohydrates. It was found that the substitution of the original hydroxy group by hydrogen or fluorine led to a narrowing of the range between the strongest and the weakest intermolecular interactions in a given structure by approximately 4 kcal/mol. The strongest interactions were found for the hydrogen bonding of hydroxy groups, followed by the hydrogen bonding of the aziridine amino group, interactions with fluorine and weak electrostatic interactions, in descending order. The intermolecular interactions that involved the fluorine atom were rather weak; however, in the structures studied they were often strengthened by other weak interactions. The fluorine atom was not able to substitute the role of the hydroxy group in the molecular packing in any of the structures. The fluorine atoms interacted solely with the hydrogen atoms located at electropositive regions of the carbohydrate molecules. A comparison of the molecular arrangements in the *exo*- and *endo*-epimines did not show a common repetitive motif, except for weak interactions below and above the parent molecule, which involved interactions of electropositive regions with pyranose and anhydro oxygen atoms. These interactions were found to be among the weakest in the structures studied, and they definitely did not determine the overall arrangement around the parent molecule. A comparison of the corresponding *exo*- and *endo*-epimines with the same C4 substituent and orientation (axial or equatorial) showed that the orientation of the aziridine group did not determine the arrangement of the molecules in the crystal lattice. Similar features were found for both the deoxygenated and the deoxyfluorinated epimines regardless of the orientation of the aziridine group. The fluorine atoms in particular were found to utilize identical interactions in the groups of C4-F_ax_ and C4-F_eq_ epimines. As a consequence, the molecular arrangement was found to be identical in both the C4-F_ax_ epimines, while in the C4-F_eq_ epimines, only the molecules near the fluorine were found in similar positions. This feature reflects the fact that interactions of single fluorine atoms are not restricted to a single molecule but are spread over at least three other molecules in the surroundings. This eventually leads to similar molecular arrangements in the structures of related compounds.

The conclusions derived from the series of 1,6-anhydro-2,3-epimino-hexopyranoses indicate the role of fluorine atoms in molecular arrangements in the crystal lattice and can help in the design of functional molecules for crystal engineering and related fields.

## Figures and Tables

**Table 1:** The difference in energy between identical molecules having NH oriented inside and outside of the molecule provided by DFT calculations.

	H	F-*cis*	F-*trans*	OH-*cis*	OH-*trans*
*exo* ΔE_out-in_ kcal/mol	1.62 (**I**)	3.31 (**II**)	0.83 (**III**)	3.88 (**IV**)	0.86 (**V**)
*endo* ΔE_out-in_ kcal/mol	0.24 (**VI**)	0.54 (**VIII**)	−0.54 (**VII**)	0.69 (**X**)	0.27 (**IX**)

**Table 2:** Hydrogen bonding in the structure of **IX**. ΔE in kcal/mol.

	OH···A (ΔE)	D···OH (ΔE)	NH···A (ΔE)	D···NH (ΔE)
**IXa**	NH_b_ (1.11)	not specific	NH_c_ (3.18)	OH_b_ (1.11)
	2.034 Å		2.439 Å	2.034 Å
**IXb**	NH_a_ (1.67)	not specific	OH_d_ (2.32)	OH_a_ (1.67)
	2.193 Å		2.394 Å	2.193 Å
**IXc**	NH_d_ (0.00)	OH_d_ (1.59)	O1_a_ (3.03)	NH_a_ (3.18)
	2.076 Å	2.216 Å	2.123 Å	2.439 Å
**IXd**	OH_c_ (1.59)	NH_b_ (2.32)	O1_b_ (2.17)	OH_c_ (0.00)
	2.216 Å	2.394 Å	2.240 Å	2.076

## Data Availability

Data available upon request.

## Supplementary Materials

The following supporting information can be downloaded. Page S2: synthesis of compound **X**. Tables SI–SX: calculated energy of interacting molecular pair., Pages S9–12: comparison of molecular arrangement in endo-epimines **VI**–**X**.
