# Supplementary material for: The Effect of Deoxyfluorination on Intermolecular Interactions in the Crystal Structures of 1,6-Anhydro-2,3-epimino-hexopyranoses
Source: Molecules. 2022 Jan 3;27(1):278. doi: 10.3390/molecules27010278 (PMC8746508; doi:10.3390/molecules27010278)
Supplement: Supplementary file 1 [file molecules-27-00278-s001.zip › molecules-1526201-supplementary.pdf]

The Effect of Deoxyfluorination on Intermolecular Interactions in the Crystal Structures of 1,6-anhydro-2,3-epimino-hexopyranoses

Martin Jakubec [1], Ivana Císařová [2], Jindřich Karban [1], Jan Sýkora [1,*]

[1] *Institute of Chemical Process Fundamentals of the Czech Academy of Sciences, v. v. i., Rozvojová 135, 165 02 Prague 6, Czech Republic;*
jakubecm@icpf.cas.cz (M.J.); karban@icpf.cas.cz (J.K.)
[2] *Department of Inorganic Chemistry, Faculty of Science, Charles University in Prague, Hlavova 2030, 128 40 Prague 2, Czech Republic; cisarova@natur.cuni.cz*
** Correspondence: sykora@icpf.cas.cz (J.S.);*

Content

1. Synthesis of Compound **X** S2

2. Tables of calculated energy of interacting molecular pairs S3

3. Comparison of Molecular Arrangement in *Endo*-epimines **VI**–**X** S9

Synthesis of compound X

General methods

Dichloromethane was dried by distillation from CaH_2 and stored over 3 Å molecular sieves, pyridine was dried by standing over NaOH. TLC was carried out with Sigma-Aldrich TLC Silica gel 60 F254 and spots were detected with an anisaldehyde solution in EtOH/AcOH/H_2SO_4. UV detection at 254 nm was also used where appropriate. Column chromatography was performed with silica gel 60 (70–230 mesh, Material Harvest). The solutions were concentrated under reduced pressure at temperatures below 45 °C. Anhydrous sodium sulfate was used to dry solutions after aqueous workup.

Scheme SI. Synthesis of compound X

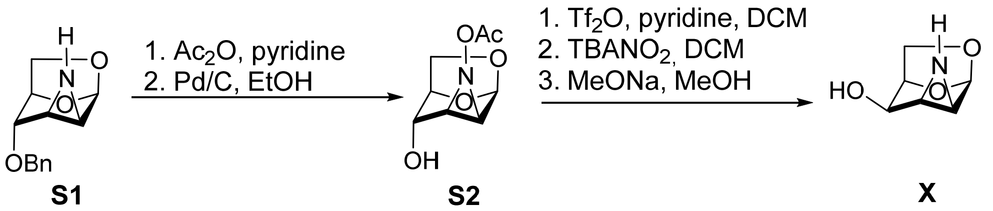

Acetic acid anhydride (100 µL, 1.06 mmol) was added to a solution of 1,6-anhydro-4-*O*-benzyl-2,3-dideoxy-2,3-epimino-β-D-mannopyranose (**S1**,[1] 192 mg, 0.82 mmol) in anhydrous pyridine (1 mL) and the resulting solution was stirred at rt overnight. The reaction mixture was then concentrated and co-evaporated with toluene (3×). The residue was dissolved in ethanol (7 mL), 10% palladium on carbon (150

mg) was added and the reaction mixture was stirred vigorously under a hydrogen-filled balloon at rt for 5 h. TLC in ethyl acetate/ethanol 10:1 showed formation of one major product (R_f 0.3). The reaction was filtered and concentrated. Chromatography of the residue in ethyl acetate/ethanol 10:1 afforded *N*-acetyl-1,6-anhydro-2,3-dideoxy-2,3-epimino-β-D-mannopyranose (**S2**, 77 mg, 47%).

Intermediate **S2** (77 mg, 0.38 mmol) was dissolved in anhydrous dichloromethane (1 mL), anhydrous pyridine was added (0.2 mL) and the resulting solution was cooled to −70 °C. Triflic anhydride (150 µL, 0.89 mmol) was added dropwise and the reaction was allowed to reach 0 °C in about 1 h. TLC in ethyl acetate/ethanol 10:1 showed formation of one major product (R_f 0.6). The reaction mixture was poured onto ice, extracted with dichloromethane (3×) and the combined extracts were dried and concentrated. The residue was dried under vacuum and dissolved in anhydrous *N*,*N*-dimethylformamide (0.5 mL) and tetrabutylammonium nitrite (0.4 g, 1.4 mmol) was added. The reaction was stirred at rt for four days. It was then loaded onto silica gel and eluted in ethyl acetate/ethanol 10:1. The fractions containing the product were collected, combined and concentrated. The residue was dissolved in 1 M sodium methanolate solution (4 mL) and stirred until the starting compound disappeared in TLC (ethyl acetate/ethanol 10:1) while the product was detected near the start. The reaction mixture was neutralized by addition of ion exchanger DOWEX 50W, filtered and concentrated to afford a syrupy residue (42 mg). Chromatography in ethyl acetate/ethanol 7:3 afforded **X** (12 mg, 22%) as a crystalline substance. Crystallization from ether provided crystals suitable for X-ray structure analysis which confirmed compound identity.

1. Karban, J.; Budesinsky, M.; Cerny, M.; Trnka, T., Synthesis and NMR spectra of 1,6-anhydro-2,3-dideoxy-2,3-epimino- and 1,6-anhydro-3,4-dideoxy-3,4-epimino-β-D-hexopyranoses. *Collect. Czech. Chem. Commun.* **2001,** *66*, 799-819.

Table SI. Calculated energy of interacting molecular pairs in **I**

pair no.	energy [a.u.]	ΔE [a.u]	ΔE[kcal/mol]
1	-878.7510444	0	0
2	-878.7455172	0.00552725	3.46834938
3	-878.7446734	0.00637099	3.99779622
4	-878.7478722	0.00317224	1.9905806
5	-878.7462727	0.00477168	2.9942292
6	-878.7430439	0.00800052	5.0203263
7	-878.7414524	0.00959197	6.01896117
8	-878.7462712	0.0047732	2.995183
9	-878.7419464	0.00909797	5.70897617
10	-878.7478701	0.00317432	1.9918858
11	-878.7510384	5.98E-06	0.00375245
12	-878.7440309	0.00701347	4.40095242
13	-878.7455186	0.00552579	3.46743322

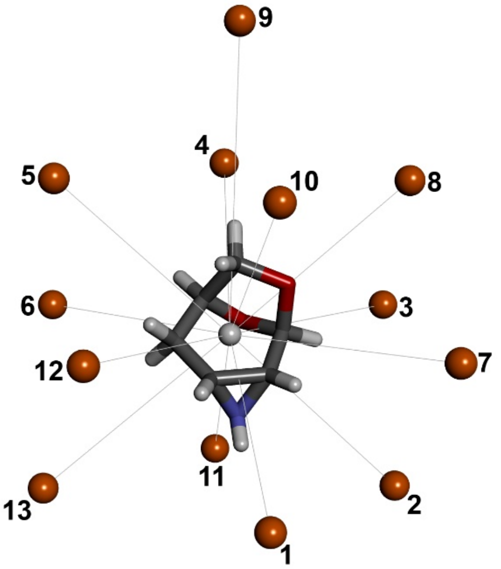

Table SII. Calculated energy of interacting molecular pairs in **II**

pair no.	energy [a.u.]	ΔE [a.u]	ΔE[kcal/mol]
1	-1077.29429409	0.00373807	2.345638925
2	-1077.29803216	0.00000000	0

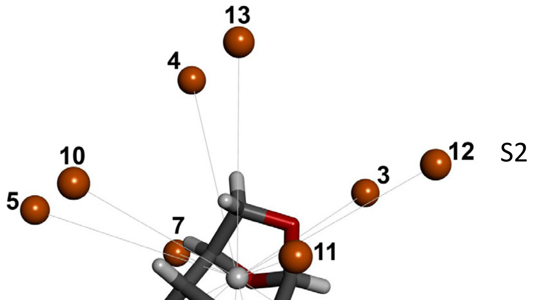

3	-1077.29802692	0.00000524	0.0032881
4	-1077.29429172	0.00374044	2.3471261
5	-1077.29746639	0.00056577	0.355020675
6	-1077.29747149	0.00056067	0.351820425
7	-1077.29418699	0.00384517	2.412844175
8	-1077.28995304	0.00807912	5.0696478
9	-1077.29617736	0.00185480	1.163887
10	-1077.29520668	0.00282548	1.7729887
11	-1077.29617554	0.00185662	1.16502905
12	-1077.29520979	0.00282237	1.771037175
13	-1077.294185	0.00384725	2.414149375

Table SIII. Calculated energy of interacting molecular pairs in **III** without (left) and with BSSE (right)

	no corrections			correction using BSSE		
pair no.	energy [a.u.]	ΔE [a.u]	ΔE[kcal/mol]	energy [a.u.]	ΔE [a.u]	ΔE[kcal/mol]
1	-1077.29402012	0.00323435	2.029554	-1077.29322187	0.00277563	1.741708
2	-1077.28936387	0.00789060	4.951351	-1077.28864346	0.00735404	4.614661
3	-1077.28916749	0.00808698	5.074580	-1077.28871112	0.00728638	4.572202
4	-1077.28917077	0.00808370	5.072522			
5	-1077.28936551	0.00788896	4.950322			
6	-1077.28986381	0.00739066	4.637639	-1077.28889994	0.00709756	4.453721
7	-1077.29725447	0.00000000	0	-1077.29599750	0.00000000	0
8	-1077.29725337	0.00000110	0.000690			
9	-1077.29275547	0.00449900	2.823123	-1077.29152892	0.00446859	2.804038
10	-1077.28976959	0.00748488	4.696762			
11	-1077.29275608	0.00449839	2.822740			
12	-1077.29402177	0.00323270	2.028519			

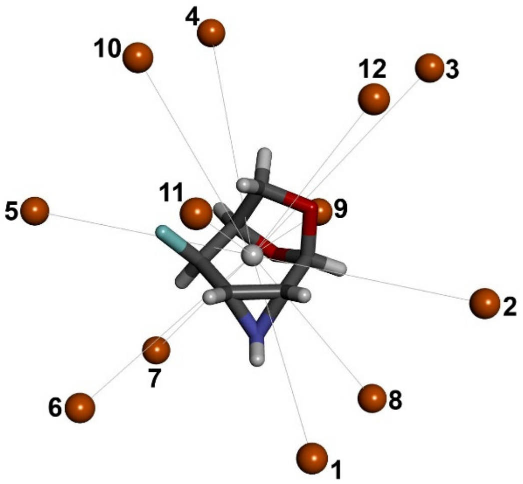

Table SIV. Calculated energy of interacting molecular pairs in **IV**

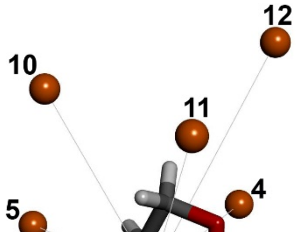

pair no.	energy [a.u.]	ΔE [a.u]	ΔE[kcal/mol]
1	-1029.24131920	0.01303608	8.1801402
2	-1029.23983301	0.01452227	9.112724425
3	-1029.24218019	0.01217509	7.639868975
4	-1029.24322928	0.01112600	6.981565
5	-1029.25435528	0.00000000	0
6	-1029.24536164	0.00899364	5.6435091
7	-1029.25435016	0.00000512	0.0032128
8	-1029.24131923	0.01303605	8.180121375
9	-1029.24218139	0.01217389	7.639115975
10	-1029.24323212	0.01112316	6.9797829
11	-1029.24536219	0.00899309	5.643163975
12	-1029.23983589	0.01451939	9.110917225

Table SV. Calculated energy of interacting molecular pairs in **V**

pair no.	energy [a.u.]	ΔE [a.u]	ΔE[kcal/mol]
1	-1029.25336581	0.00000000	0
2	-1029.24027862	0.01308719	8.212211725
3	-1029.24387852	0.00948729	5.953274475
4	-1029.25336371	0.00000210	0.00131775
5	-1029.24027837	0.01308744	8.2123686
6	-1029.24388050	0.00948531	5.952032025
7	-1029.24468881	0.00867700	5.4448175
8	-1029.24321170	0.01015411	6.371704025
9	-1029,24407437	0,00929144	5,8303786
10	-1029.24071983	0.01264598	7.93535245
11	-1029.24470797	0.00865784	5.4327946
12	-1029.24321312	0.01015269	6.370812975
13	-1029.24407696	0.00928885	5.828753375
14	-1029.24072	0.01264543	7.935007325

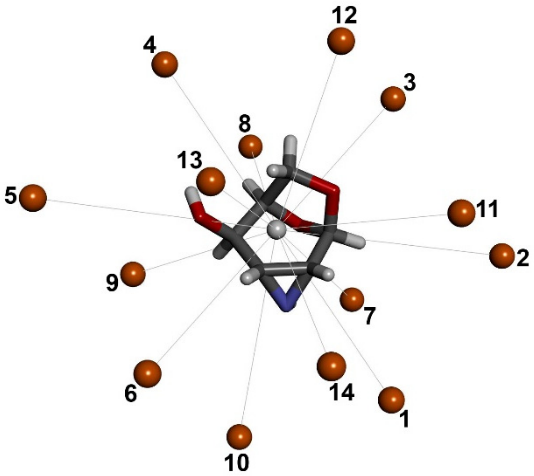

Table SVI. Calculated energy of interacting molecular pairs in **VIa** (left) and **VIb** (left)

pair no.	energy [a.u.]	ΔE [a.u]	ΔE[kcal/mol]
1	-878.74470399	0.00000384	0.0024096
2	-878.74082860	0.00387923	2.434216825
3	-878.74308608	0.00162175	1.017648125
4	-878.74330265	0.00140518	0.88175045
5	-878.73953871	0.00516912	3.2436228
6	-878.74032095	0.00438688	2.7527672
7	-878.74330708	0.00140075	0.878970625
8	-878.74159584	0.00311199	1.952773725
9	-878.74044282	0.00426501	2.676293775

pair no.	energy [a.u.]	ΔE [a.u]	ΔE[kcal/mol]
1	-878.74399946	0.00070837	0.444502
2	-878.74278765	0.00192018	1.204913
3	-878.74082992	0.00387791	2.433389
4	-878.74120803	0.00349980	2.196124
5	-878.74159584	0.00311199	1.952774
6	-878.73956465	0.00514318	3.227345
7	-878.74470783	0.00000000	0
8	-878.73717058	0.00753725	4.729624
9	-878.74120838	0.00349945	2.195905

10	-878.74032003	0.00438780	2.7533445
11	-878.74044127	0.00426656	2.6772664
12	-878.74400239	0.00070544	0.4426636

10	-878.74308528	0.00162255	1.01815
11	-878.74279017	0.00191766	1.203332
12	-878.73716731	0.00754052	4.731676

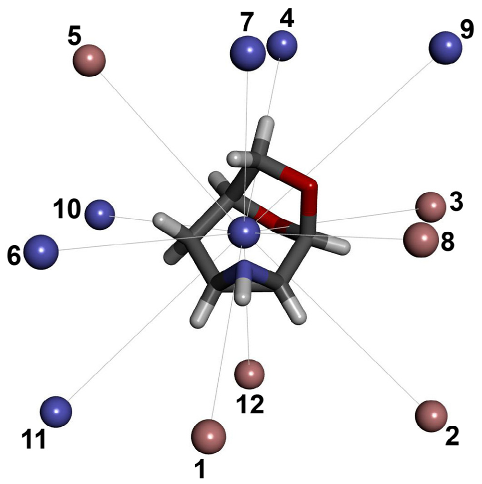

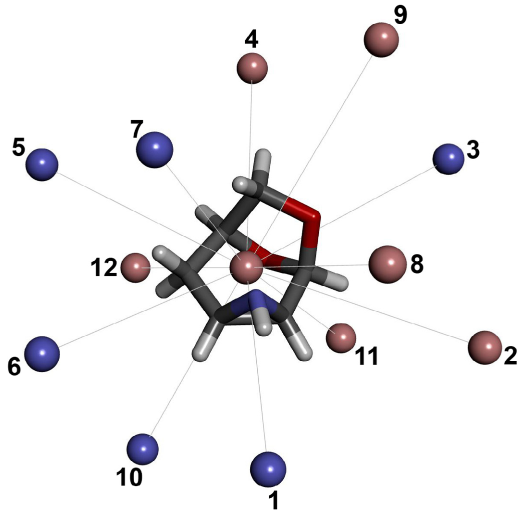

Table SVII. Calculated energy of interacting molecular pairs in **VII**

pair no.	energy [a.u.]	ΔE [a.u]	ΔE[kcal/mol]
1	-1077.28864765	0.00000000	0
2	-1077.28656193	0.00208572	1.308789
3	-1077.28310568	0.00554197	3.477586
4	-1077.28680196	0.00184569	1.15817
5	-1077.28609760	0.00255005	1.600156
6	-1077.28656502	0.00208263	1.30685
7	-1077.28152307	0.00712458	4.470674
8	-1077.28680430	0.00184335	1.156702
9	-1077.28610188	0.00254577	1.597471
10	-1077.28151832	0.00712933	4.473655
11	-1077.28310990	0.00553775	3.474938
12	-1077.28864501	0.00000264	0.001657

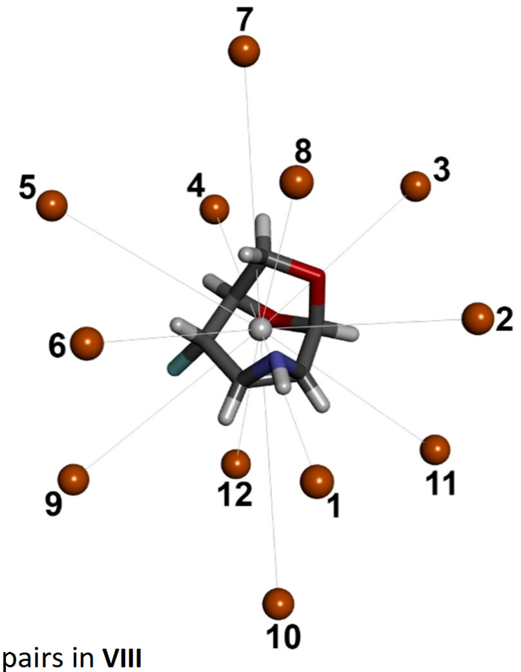

Table SVIII. Calculated energy of interacting molecular pairs in **VIII**

pair no.	energy [a.u.]	ΔE [a.u]	ΔE[kcal/mol]
1	-1077.28863260	0.00000000	0
2	-1077.28194591	0.00668669	4.19589797
3	-1077.28863127	0.00000133	0.00083458
4	-1077.28608471	0.00254789	1.59880097
5	-1077.28194586	0.00668674	4.19592935
6	-1077.28527346	0.00335914	2.10786035
7	-1077.28125922	0.00737338	4.62679595
8	-1077.28607962	0.00255298	1.60199495
9	-1077.28250155	0.00613105	3.84723388

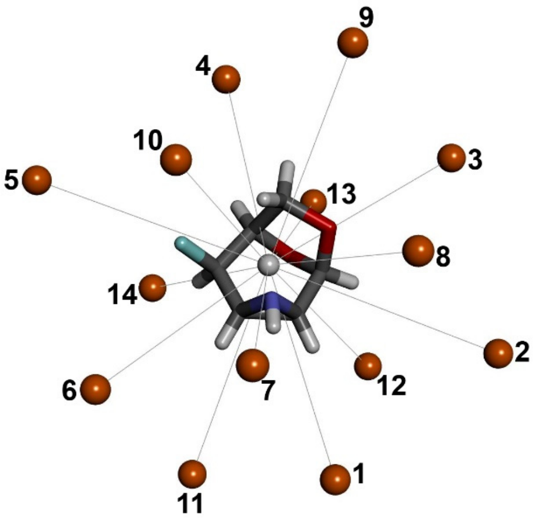

10	-1077.28719038	0.00144222	0.90499305
11	-1077.28250399	0.00612861	3.84570278
12	-1077.28527520	0.00335740	2.1067685
13	-1077.28125788	0.00737472	4.6276368
14	-1077.287193	0.00143937	0.90320468

Table SIXa. Calculated energy of interacting molecular pairs in **IXa** (left) and **IXb** (right)

pair no.	energy [a.u.]	ΔE [a.u]	ΔE[kcal/mol]	pair no.	energy [a.u.]	ΔE [a.u]	ΔE[kcal/mol]
1	-1029.23194837	0.00507050	3.18173875	1	-1029.23332281	0.00369606	2.31927765
2	-1029.22659582	0.01042305	6.540463875	2	-1029.22373002	0.01328885	8.338753375
3	-1029.22677474	0.01024413	6.428191575	3	-1029.23048888	0.00652999	4.097568725
4	-1029.22372412	0.01329475	8.342455625	4	-1029.22990587	0.00711300	4.4634075
5	-1029.22729875	0.00972012	6.0993753	5	-1029.22937134	0.00764753	4.798825075
6	-1029.23027444	0.00674443	4.232129825	6	-1029.22965845	0.00736042	4.61866355
7	-1029.22574956	0.01126931	7.071492025	7	-1029.23526098	0.00175789	1.103075975
8	-1029.23435892	0.00265995	1.669118625	8	-1029.22660267	0.01041620	6.5361655
9	-1029.22662836	0.01039051	6.520045025	9	-1029.22718709	0.00983178	6.16944195
10	-1029.22677268	0.01024619	6.429484225	10	-1029.23206172	0.00495715	3.110611625
11	-1029.23218918	0.00482969	3.030630475	11	-1029.23356640	0.00345247	2.166424925
12	-1029.23525638	0.00176249	1.105962475	12	-1029.23436282	0.00265605	1.666671375

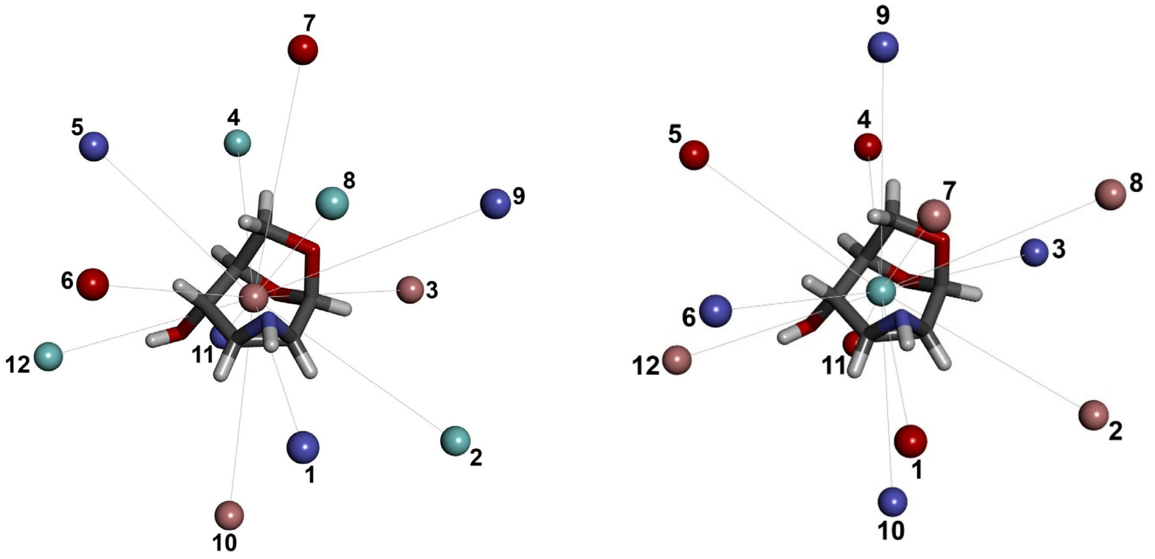

Table SIXb. Calculated energy of interacting molecular pairs in **IXc** (left) **IXd** (right)

pair no.	energy [a.u.]	ΔE [a.u]	ΔE[kcal/mol]	pair no.	energy [a.u.]	ΔE [a.u]	ΔE[kcal/mol]
1	-1029.23218918	0.00482969	3.030630475	1	-1029.23356678	0.00345209	2.166186475
2	-1029.22662815	0.01039072	6.5201768	2	-1029.22754091	0.00947796	5.9474199
3	-1029.23206707	0.00495180	3.1072545	3	-1029.23027638	0.00674249	4.230912475
4	-1029.22729509	0.00972378	6.10167195	4	-1029.23007327	0.00694560	4.358364

5	-1029.23007365	0.00694522	4.35812555
6	-1029.23038686	0.00663201	4.161586275
7	-1029.22965690	0.00736197	4.619636175
8	-1029.23195100	0.00506787	3.180088425
9	-1029.22754418	0.00947469	5.945367975
10	-1029,23450025	0,00251862	1,58043405
11	-1029.23701746	0.00000141	0.000884775
12	-1029.22718266	0.00983621	6.172221775

5	-1029.22990404	0.00711483	4.464555825
6	-1029.23006954	0.00694933	4.360704575
7	-1029.23006994	0.00694893	4.360453575
8	-1029.23701887	0.00000000	0
9	-1029.22937420	0.00764467	4.797030425
10	-1029.23331380	0.00370507	2.324931425
11	-1029.23449128	0.00252759	1.586062725
12	-1029.22575177	0.01126710	7.07010525

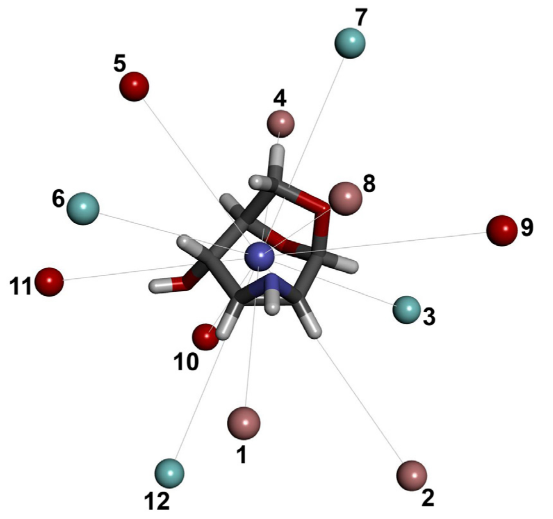

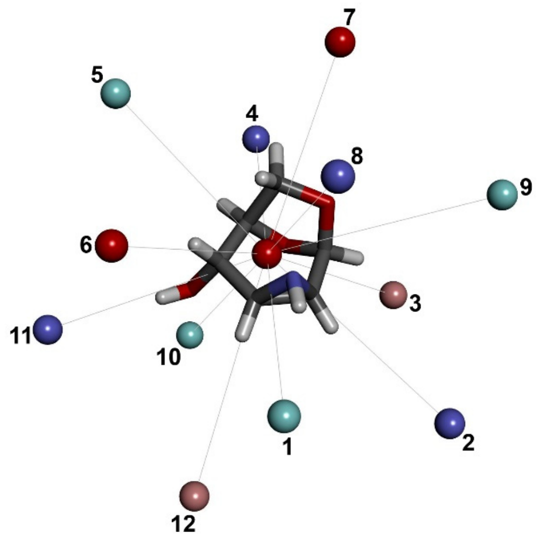

Table SX. Calculated energy of interacting molecular pairs in **X**

pair no.	energy [a.u.]	ΔE [a.u]	ΔE[kcal/mol]
1	-1029.23404780	0.00853651	5.356660025
2	-1029.23208146	0.01050285	6.590538375
3	-1029.22951861	0.01306570	8.19872675
4	-1029.23343593	0.00914838	5.74060845
5	-1029.24258431	0.00000000	0
6	-1029.22992432	0.01265999	7.944143725
7	-1029.22748867	0.01509564	9.4725141
8	-1029.23404767	0.00853664	5.3567416
9	-1029.22951895	0.01306536	8.1985134
10	-1029.24258048	0.00000383	0.002403325
11	-1029.22992538	0.01265893	7.943478575
12	-1029.23208038	0.01050393	6.591216075
13	-1029.22749147	0.01509284	9.4707571
14	-1029.233438	0.00914615	5.739209125

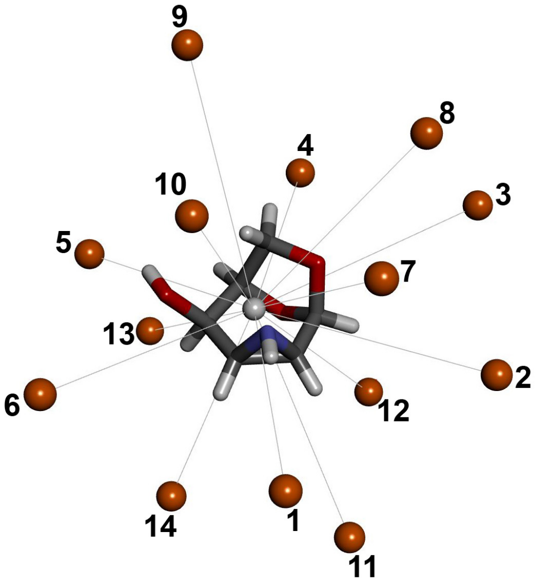

Comparison of Molecular Arrangement in *Endo*-epimines **VI**–**X**

A similar packing motif was found in the surroundings of unsubstituted molecule **VIa** and *ax*-fluoro epimine **VII** (Figure S1). The common interaction was found for the molecule approaching the parent molecule from the top. The joint interactions involve hydrogen at C5 of the molecule approaching from above and anhydro oxygen of the parent molecule and reversed interaction of C6 hydrogen with the pyranose oxygen. In **VIa**, this double interaction is further supported by the interaction of C6 hydrogen with the nitrogen lone pair of the parent molecule (ΔE=0.88 kcal/mol, molecule no 7). In **VII**, the approaching molecule is slightly shifted to the front, away from the nitrogen reach (ΔE=1.16 kcal/mol, molecule no 8). The pairs with the same interactions and overall energy are generated in both structures by symmetry operations with the role of the parent and approaching molecule interchanged (molecule no. 4 in both cases). These molecules are mutually more shifted in space, and the differences in interactions are more pronounced there. Furthermore, there is another interaction common to both structures where molecule no. 12 approaches the parent molecule from the bottom with the NH group interacting with its pyranose oxygen. This is a very strong interaction—the strongest in **VII** and the second strongest in **VIa** (ΔE=0.44 kcal/mol). The same interaction in reverse is utilized by molecule no. 1 in both structures. In the case of **VIa**, the molecules interacting via NH hydrogen bonding are both generated from molecule **VIb**, which results in slightly different positioning and orientation when compared to the packing of **VII**.

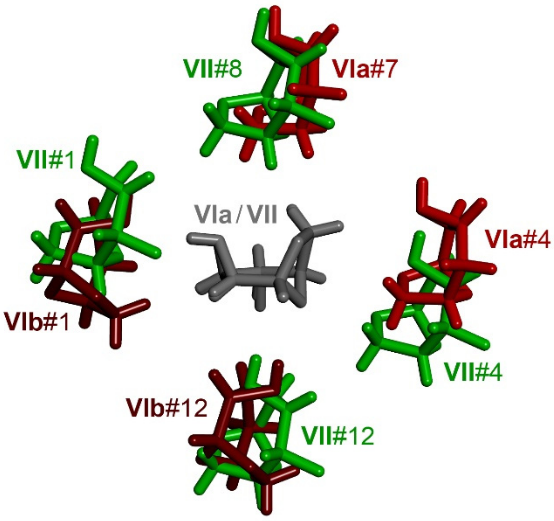

Figure S1. An overlay of similar segments in molecular packing of **VIa** (red) and **VII** (green). Parent molecules are depicted in grey, molecules **VIb** in dark red.

The structures of fluoro epimines **VII** and **VIII** have completely different molecular packing from each other except for the molecules above and below the parent molecule, which are both generated by translation. The mutual interaction between parent and approaching molecule is among the weakest ones in the given structure. Obviously, the position and orientation of the approaching molecule is determined by interactions with other molecules rather than with the parent molecule. The direction of the translation is mutually tilted in both structures reflecting different positions of all molecules in both structures (Figure S2).

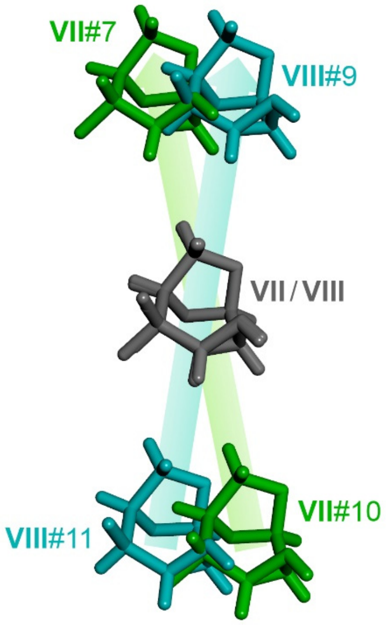

Figure S2. An overlay of molecules generated by translation in molecular packing of **VII** (green) and **VIII** (turquoise). Parent molecules are depicted in grey. The directions of the translation in individual packings are indicated by arrows.

The structure of fluoro epimine **VII** also shows a similar interaction motif to one molecule of hydroxy epimine **IX**. Although the substitution in both epimines is in axial position and though structure **IX** consists of four independent molecules, the packing similarity is restricted solely to molecule no. 7 in the surroundings of **IXa**, which is located above the parent molecule (Figure S3). Molecule no. 7 is in both structures slightly shifted. No significant interaction with the parent molecules is apparent. DFT calculations identified these pairs as among the weakest in both structures. Both molecules are probably placed in a similar position just accidentally, as for **VII** and **VIII** discussed above. In **VII**, molecule no. 7 is generated from the parent molecule by translation. The same operation generates molecule no. 10 in **VII**, while in **IX**, both positions are occupied by molecules of different origin, **IXd** and **IXa**, respectively, and therefore of different mutual orientation.

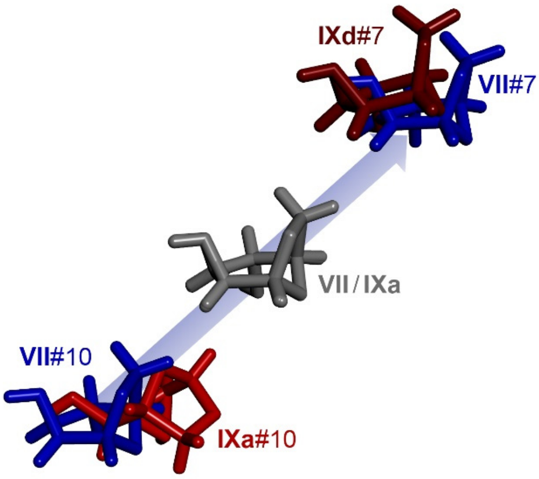

Figure S3. An overlay of similar packing motifs in structure **VII** (blue) and **IXa** (red). Parent molecules are depicted in grey, molecule **IXd** in dark red. The direction of the translation is indicated by the blue arrow.

Molecules no. 9 and 11, which are associated with the parent molecule in fluoro epimine **VIII** by translation, as discussed above, are also localized in similar positions and orientation as some molecules in the packing of hydroxy epimine **IX**. The similarity was found in the surroundings of all four molecules forming the independent part of the unit cell in **IX**. Interestingly, in **IXa** and **IXb**, only the upper molecule overlaps with the upper molecule in **VIII**, while **IXc** and **IXd** coincide with the molecule below the parent molecule. Despite appearances, the overlapping molecules in the surroundings of **IXa**–**d** were not generated by translation from the parent molecule. Actually, the overlapping molecules are generated by symmetry operations from different independent molecules in **IX**. The molecule in **IXa** corresponds to **IXd** and vice versa, and that in **IXb** corresponds to **IXc** and the other way around (Figure S4). Again, the interactions of the molecules discussed with the parent molecule are very weak; therefore, their positions and orientation are determined by interaction with other molecules rather than with the parent molecule.

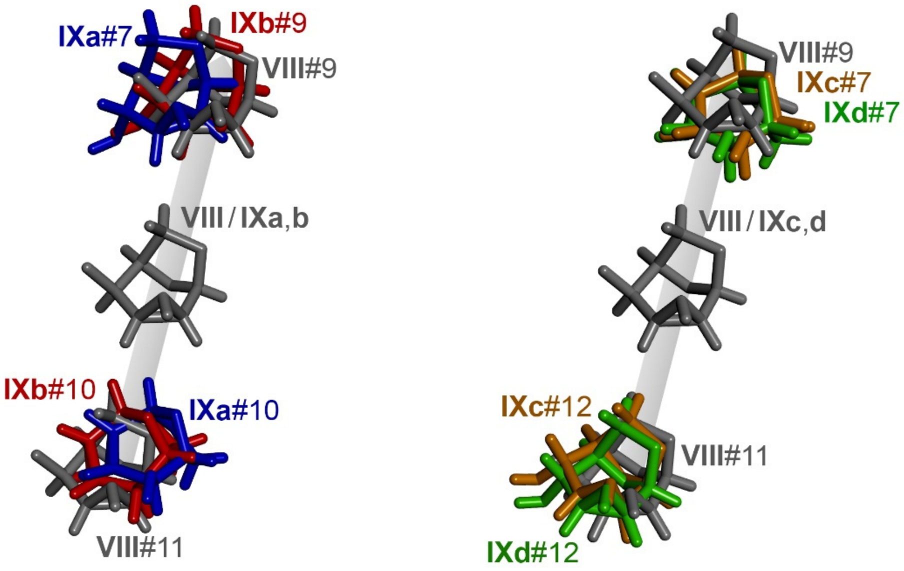

Figure S4. An overlay of similar packing motifs in structure **VIII** (grey) and **IX** (**IXa** blue, **IXb** red, **IXc** gold, **IXd** green). Parent molecules are depicted in grey. The direction of the translation in **VIII** is indicated by the grey arrow.

A significant overlap in molecular packings was found in the surroundings of molecules **VIb** and **X**. In these structures, four molecules are in very close positions, while the packing of the rest is completely different. Two of the molecules of interest (e.g. molecules no. 2 and 12 in **X**) interact with the parent molecule by a rather strong interaction of hydrogen at C1 with the pyranose oxygen. These molecules were generated by symmetry operation, they are mutually related by translation and their role in the interaction towards parent molecule is reversed (Figure S5). The other two (e.g. molecules no. 7 and 13 in **X**) were generated from the parent molecule by translation, and there is no obvious interaction with the parent molecule. On the other hand, the latter molecules are tied with the other two (molecules no. 2 and 12 in **X**) again by interaction of hydrogen at C1 with the pyranose oxygen. The energy difference between both types of interaction toward parent molecules (i.e. interaction of molecules no. 2 and 12 versus molecules no. 7 and 13 in **X**) is 3.5 kcal/mol in **VIb** and 2.9 kcal/mol in **X**. It is worth mentioning that all discussed molecules in the surroundings of **VIb** originate from the parent molecule (**VIb**).

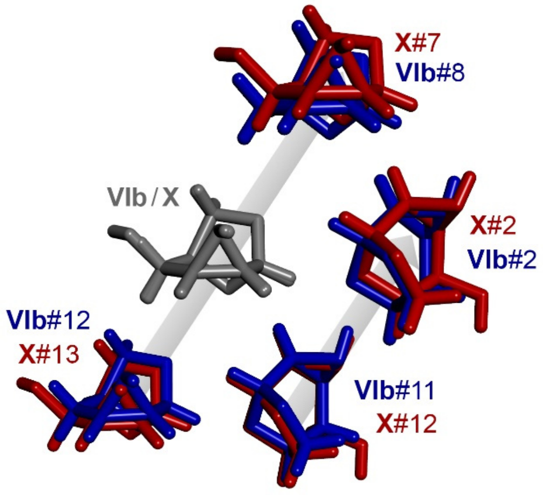

Figure S5. An overlay of similar packing motifs found in structure **VIb** (blue) and **X (**red). Parent molecules are depicted in grey. The direction of the translation is indicated by grey arrows.

The fluorine atom was not able to deputize the role of the hydroxy group in the molecular packing in any of the structures. Thus no similarities were found between structures with the same orientation of fluorine and hydroxy group in *exo-* and *endo*-epimines (**II** and **IV**, **III** and **V**, **VII** and **IX**, and **VIII** and **X**).
